# Managing hydrocephalus in 54 infants under 3 months of age: A single center cohort study

**DOI:** 10.1007/s00381-025-06769-6

**Published:** 2025-02-18

**Authors:** S. R. M. Van Rijen, F. Groenendaal, K. S. Han, M. L. Tataranno, P. A. Woerdeman

**Affiliations:** 1 Division of Neuroscience, Department of Neurosurgery, University Medical Center Utrecht, and Utrecht University, PO Box 85500, 3508 GA Utrecht, The Netherlands; 2 Department of Neonatology, Wilhelmina Children’s Hospital, University Medical Center Utrecht, and Utrecht University, Utrecht, The Netherlands

**Keywords:** Hydrocephalus, Endoscopic third ventriculostomy, Ventriculoperitoneal shunt, Neonatal, Ventricular access device

## Abstract

**Purpose:**

Managing hydrocephalus in infants can be very challenging. The most used permanent hydrocephalus treatment in young patients is a ventriculoperitoneal shunt (VPS) placement. Obstructive hydrocephalus in selected young patients can be treated with endoscopic third ventriculostomy (ETV). However, in infants less than 6 months of age, the outcome of both procedures remains to be bothered with complications, revision surgeries and long-term shunt dependency. This retrospective study analyzes the management of hydrocephalus in 54 very young infants with different etiological causes.

**Methods:**

Data was collected retrospectively from a single center university hospital over a 5-year period (2018–2022). All patients under 3 months of age with progressive ventriculomegaly confirmed by cranial ultrasound (cUS), who required neurosurgical intervention, were eligible for this study. Hydrocephalus was treated with serial tapping from a ventricular access device (VAD), placement of ventriculoperitoneal shunts and/or performing a thulium laser-assisted ETV.

**Results:**

Twelve patients benefited sufficiently from a VAD to normalize ventricular volume lastingly. Forty-two patients required permanent treatment (28 underwent a VPS; 14 an ETV if there was obstructive hydrocephalus) at an average age of 2.5 months. The VPS failure rate was 32.1% and the ETV failure rate was 50%. Although not significantly different, patients with failed ETV tended to be younger than patients with successful ETV (*p* = 0.38). One week before permanent ETV treatment, relatively large ventricular volumes were measured in failed ETV patients, as compared to successful ETVs.

**Conclusions:**

Managing hydrocephalus in very young infants remains challenging regarding surgical strategy, reducing shunt dependency and decreasing current complication rates. In addition to a VPS, an ETV has shown to be a successful treatment option for hydrocephalus in well-selected very young infants. The opportunity to decrease ventricular volume with a VAD could have contributed to the success of an ETV in this young patient group.

## Introduction

Hydrocephalus is a common neurological condition in infants [1]. It is characterized by excessive accumulation of cerebrospinal fluid (CSF), leading to enlarged ventricles generally followed by increased intracranial pressure (ICP). This may negatively affect the brain and can finally lead to brain parenchymal atrophy. Currently, in young patients, the most used permanent hydrocephalus treatment is a ventriculoperitoneal shunt (VPS) placement. Obstructive hydrocephalus in selected young patients can be treated with endoscopic third ventriculostomy (ETV) [2–6]. With the development of endoscopic techniques, ETV has become an important alternative to shunting [1, 7]. However, in infants less than 6 months old, the outcome of both procedures remains to be bothered with complications and adverse sequelae. Especially very young infants and premature neonates score relatively high rates of complications after shunt operations including infection, obstruction, over drainage of CSF or hemorrhage [8, 9]. Nevertheless, in addition to the etiology of hydrocephalus and whether a shunt has been placed in the past, age in particular appears to be an important predictor for the ETV success score (ETVSS) with high failure rates reported in neonates and young infants less than 6 months old ranging from 55.3% to 83.5% [2, 3, 7, 9–14]. The aim of this retrospective study was to analyze the management of hydrocephalus in 54 very young infants with different etiologies.

## Methods

### In- and exclusion criteria

A retrospective cohort study was performed at the Wilhelmina Children’s Hospital (WKZ) over a period of 5 years (2018–2022). The WKZ is part of the University Medical Center Utrecht and serves as a high-volume referral center for Neonatology and Pediatric Neurosurgery in the Netherlands.

For this study, all patients under 3 months of age (patients age is uncorrected for the postnatal age) with progressive ventriculomegaly in the neonatal period confirmed by cranial ultrasound (cUS) who required neurosurgical intervention were eligible. Only patients who had at least 6 months follow-up after last performed procedure were included. Patients in whom the hydrocephalus appeared to be based on a brain tumor were excluded.

### Hydrocephalus treatment procedure

At our center, neurosurgical interventions are often initiated with the placement of a ventricular access device (VAD) for CSF volume temporization when refractory to three CSF volume temporizing lumbar punctures. Especially in preterm infants with posthemorraghic ventricular dilatation, a ventricular access device to tap the ventricles is often used as a first neurosurgical procedure due to low weight, clinical condition, high concentrations of blood products and proteins in the CSF, and the abdomen not being suitable for a shunt system at that moment in time [15]. Such a device provides an easy access for CSF aspiration, pressure measurement, and diagnostic purposes including laboratory results of CSF protein and erythrocyte concentrations. Patients were eligible for permanent neurosurgical treatment if they subsequently remained VAD puncture dependent after 4–6 weeks with persistently dilated ventricles on cUS. Magnetic Resonance Imaging (MRI) was performed prior to permanent treatment to differentiate between obstructive components in the CSF pathway versus communicating hydrocephalus. If there were signs of radiological obstruction sites, both VPS and ETV as permanent treatment were discussed in detail with the parents and a shared decision on surgical treatment was made. A shunt placement was done in a communicating hydrocephalus.

A weight of at least 2000 g and low CSF concentrations of erythrocytes (< 100/mm3) and proteins (< 1.5 g/L) were required prior to the permanent treatment [16]. In VPS procedures, the proximal catheter preferred position was frontal, using the existing VAD pathway. The ETV procedures were performed as described by Boorder et al., using a thulium laser with energy settings ≤ 2W [17]. If a VAD was in situ, it was replaced after performing the ventriculostomy.

### Collected data

Data regarding the patients were collected retrospectively from patients’ files including birth weight, weeks of gestation, age at time of VAD placement, days of VAD punctures, hydrocephalus etiology (intraventricular hemorrhage (IVH), aqueduct stenosis, craniocervical stenosis, spina bifida with Chiari malformation, infection, congenital and intracranial cyst), IVH classification, days to last follow-up examination and the Bayley Scales of Infant and Toddler Development-Third Edition-Dutch version (BSID-III-NL) assessment tool, need for permanent treatment including VPS or ETV, age at time of permanent treatment, failure of permanent treatment, complications < 24 h and < 30 days after performing the VAD placement, VPS or ETV procedure, number of procedures per patients, ventricular measurements (ventricular index (VI), anterior horn width (AHW), thalamo-occipital distance (TOD)), CSF values (number of erythrocytes and proteins) and the ETVSS. Signed parental informed consent for this study was obtained in all cases. Data collection adhered to research ethics protocols.

The ventricular measurements were obtained from cUS. VI was measured as described by Levene [18] and both AHW and TOD as described by Davies et al. [19]. For this research, we used ventricular measurement and CSF values of erythrocytes and proteins within one week before permanent treatment. The severity of IVH is described according to the widely used classification [20].

ETV failure was defined as the requirement of a VPS for CSF diversion to manage hydrocephalus in patients with signs and/or symptoms of raised ICP and confirmed dilated ventricles on imaging (MRI/cUS). VPS failure was defined as any subsequent surgical procedure (including VPS revisions and/or conversion to an ETV) for definitive CSF diversion in the treatment of hydrocephalus in patients with confirmed dilated ventricles on imaging and signs and/or symptoms of raised ICP.

### Data analysis

Data were analyzed using IBM SPSS Statistics, version 27 (IBM Corp.). Categorial variables were presented as numbers and percentages. Continuous variables were presented as the mean (± Standard Deviation (SD)). *P* values were adjusted for multiple comparisons with the use of an independent t-test. Univariate linear regression was used to correct for possible confounding. Statistical significance was set at *p* < 0.05.

## Results

In a total of 54 included patients, 128 procedures for the treatment of hydrocephalus were performed. In 48 patients (88.9%), the initial treatment consisted of placing a VAD. In the remaining 6 patients (11.1%), a direct permanent treatment was done (3 received VPS treatment; 3 an ETV). Of 48 patients who initially received a VAD, 12 benefited sufficiently from the VAD and did not require any other neurosurgical treatment. The hydrocephalus etiology of all these 12 patients was IVH. The remaining 36 patients had to undergo permanent treatment of whom 25 were treated with a VPS and 11 with an ETV.

**Fig. 1:**
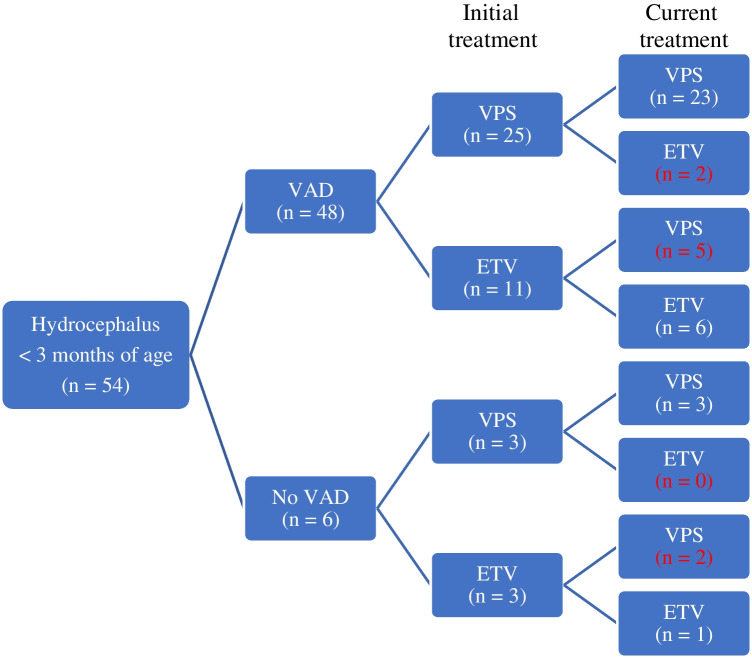
Flow diagram of number of patients with and without VAD, initial permanent treatment and current treatment. In red the number of patients with failed initial treatment converted to new type of treatment. *VAD ventricular access device; VPS ventriculoperitoneal shunt; ETV endoscopic third ventriculostomy*

In total, 42 patients (with or without VAD in history) who required permanent treatment, a VPS was needed in 28 patients and an ETV in 14 patients (Fig. 1).

Table 1 shows patients’ characteristics of the study population. The mean birth weight was 2275 g (range 680 g to 4200 g). 35 patients (64.8%) were born prematurely. The mean gestational age was 34 weeks (range 25 to 42 weeks).

**Table 1:** Patients’ characteristics of study population (*n* = 54)

	VAD only*	VPS	ETV	Total	*p* value VPS VS ETV (95% CI)
Patients n (%)	12 (22.2)	28 (51.9)	14 (25.9)	54	
Birth weight in grams, mean (SD)	1824 (778)	2363 (975)	2485 (1075)	2275 (978)	0.71 (−790 – 545)
Premature n (%)	10 (28.6)	17 (48.6)	8 (22.8)	35	
Weeks of gestation, mean (SD)	31 (4)	35 (5)	35 (5)	34 (5)	0.88 (−3 – 3)
age at time of VAD placement in days, mean (SD)	19 (12)	29 (29)	25 (31)	25 (26)	0.71 (−18 – 25)
Corrected age at time of VAD placement in weeks, mean (SD)	34 (5)	38 (6)	38 (4)	37 (5)	0.97 (−4 – 4)
CSF aspiration via VAD in days, mean (SD)	24 (21)	42 (20)	39 (13)	37 (20)	0.72 (−11 – 16)
Etiology HC *n* (%)					
IVH	12 (41.4)	17 (58.6)	0 (0.0)	29	
IVH + stenosis	0 (0.0)	2 (22.2)	7 (77.8)	9	
IVH + infection	0 (0.0)	2 (66.7)	1 (33.3)	3	
Stenosis of cerebral aqueduct	0 (0.0)	1 (16.7)	5 (83.3)	6	
Craniocervical stenosis	0 (0.0)	1 (100.0)	0 (0.0)	1	
Spina bifida aperta with Chiari malformation	0 (0.0)	2 (100.0)	0 (0.0)	2	
Infection	0 (0.0)	1 (100.0)	0 (0.0)	1	
Congenital	0 (0.0)	1 (100.0)	0 (0.0)	1	
Cyst	0 (0.0)	1 (50.0)	1 (50.0)	2	
IVH classification n (%)					
Grade 2	6 (50.0)	4 (33.3)	2 (16.7)	12	
Grade 3	2 (18.2)	7 (63.6)	2 (18.2)	11	
PVHI	1 (20.0)	4 (80.0)	0 (0.0)	5	
HC imaging characteristics					
Obstructive n (%)	0 (0.0)	12 (46.2)	14 (53.8)	26	
Communicating n (%)	12 (42.9)	16 (57.1)	0 (0.0)	28	
Follow-up period in days, mean (SD)	865 (458)	1234 (579)	944 (306)	1077 (515)	0.09 (−45 – 626)

^*^VAD only defined as: until patient was no longer dependent on CSF punctures with stable ventricular volume and clinical assessment

*VAD* ventricular access device, *VPS* ventriculoperitoneal shunt, *ETV* endoscopic third ventriculostomy, *CI* confidence interval, *SD* standard deviation, *CSF* cerebrospinal fluid, *HC* hydrocephalus, *IVH* intraventricular hemorrhage, *PVHI* periventricular hemorrhagic infarction

The mean age at time of VAD placement was 25 days not corrected for prematurity (range 1 to 104 days). The corrected age of time at VAD placement was 37 weeks (range 28 to 51), with a mean of 37 CSF aspiration days via VAD (range 4 to 83 days). The mean weight at time of VAD placement was 1943 g (range 990 g to 3900 g) in patients who only received a VAD. In the VPS group the mean weight at time of VAD placement was 2667 g (range 940 g to 4480 g) and 2684 g (range 1090 g to 3762 g) in the ETV group.

The etiology of hydrocephalus was as follows: 29 IVH (53.7%), 9 IVH with stenosis (16.7%), 3 IVH with subsequent infection (5.6%), 6 congenital aqueduct stenosis (11.1%), 1 craniocervical stenosis (1.9%), 2 spina bifida aperta with Chiari malformation (3.7%) 1 infection (1.9%), 1 congenital malformation (Dandy Walker malformation) (1.9%) and 2 subarachnoidal cysts (3.7%).

All 5 patients with periventricular hemorrhagic infarction (PVHI) also had an IVH grade 3.

Among the 54 included patients, 26 had an obstructive hydrocephalus (48.1%) (12 treated with a VPS, 14 with an ETV) and 28 had a communicating hydrocephalus (51,9%) (16 treated with a VPS, none with an ETV, the other 12 benefited sufficiently from the VAD CSF tapping period). The mean follow-up period was 1077 days.

### VPS

28 patients received a VPS as initial permanent treatment of whom 3 patients did not receive a VAD prior to the VPS because of sufficient body weight and relatively late diagnosis of hydrocephalus which was the case in 2 patients with spina bifida aperta with Chiari malformation and 1 patient with craniocervical stenosis.

In this group the mean gestational age was 35 weeks (SD 5), with 17 patients prematurely born. The hydrocephalus etiology of these 28 patients was 17 patients with IVH, 2 with IVH and aqueduct stenosis, 2 with IVH combined with an infection (1 E. coli meningitis and 1 candida meningitis), 1 congenital aqueduct stenosis, 1 craniocervical stenosis, 2 spina bifida aperta with Chiari malformation, 1 infection (toxoplasmosis ventriculitis), 1 congenital cause (Dandy Walker malformation) and 1 interhemispheric cyst with compression on the aqueduct. Twelve patients had an obstructive component in the ventricular pathways detected on MRI prior to permanent treatment. The average follow-up period consisted of 1234 days (SD 579).

The mean age at the time of permanent treatment was 73 days. The mean corrected age at time of permanent treatment was 44 weeks. In 9 of 28 patients (32.1%), the VPS failed. In 2 patients the VPS was converted to an ETV (both patients had aqueductal stenosis). VPS revisions were needed in 9 patients. On average, VPS failure was diagnosed at 146 days. A median of 2 operations were done per patient in successful shunted patients. A median of 3 surgeries were needed when a shunt failed. (Table 2).

**Table 2:** Permanent treatment

	**VPS**	**ETV**	**Total**	***p* value (95% CI)**
Number of patients with first procedure, *n* (%)	28 (66.7)	14 (33.3)	42	
Treatment age in days, mean (SD)	73 (42)	77 (60)	75 (48)	0.82 (−36 – 28)
Corrected treatment age in weeks, mean (SD)	44 (5)	46 (7)	45 (6)	0.45 (−5 – 2)
Failure rate, *n* (%)	9 (32.1)	7 (50.0)	16	
Period until failure in days, mean (SD)	146 (194)	78 (80)	116 (155)	0.40 (−101 – 236)
Current treatment, *n* (%)	33 (78.6)	9 (21.4)	42	
Complications < 24 h, *n* (%)	2 (3.4)	0 (0.0)	3	
Complications < 30 days, *n* (%)	2 (3.4)	1 (6.3)	3	
Number of procedures per patient with successful treatment, median (IQR)	2 (2–2)	2 (2–2)		
Number of procedures per patient with failed treatment, median (IQR)	3 (3–5)	4 (2–5)		

*VPS* ventriculoperitoneal shunt, *ETV* endoscopic third ventriculostomy, *CI* confidence interval, *SD* standard deviation, *IQR* interquartile range

### ETV

14 patients received an ETV as initial permanent treatment of whom 3 patients did not receive a VAD prior to the ETV which was the case in 2 patients with aqueduct stenosis and 1 patient with IVH with stenosis.

They had a mean birthweight of 2485 g. The mean gestational age was 35 weeks. Eight patients were prematurely born. The mean age at time of VAD placement was 25 days with an average of 39 days for temporizing CSF volume until permanent treatment with ETV appeared to be necessary. The etiology consisted of 7 patients with IVH and associated stenosis (including 5 with an aqueduct stenosis, 1 with a trapped ventricle and 1 with a Monro block), 1 IVH combined with infection (candida meningitis), 5 aqueduct stenosis and 1 interhemispheric cyst with compression on the aqueduct. The average follow-up period was 944 days (SD 306).

The mean age at time of permanent treatment was 77 days. The mean corrected age at time of permanent treatment was 46 weeks. In 7 patients (50%), the ETV failed and had to be converted to a VPS. The hydrocephalus etiology of these patients consisted of 3 IVH with aqueduct stenosis, 2 aqueduct stenosis, 1 IVH combined with infection (candida meningitis) and 1 interhemispheric cyst with compression on the aqueduct. On average, ETV failed within 78 days. A median of 2 surgeries were done per patient in successful ETVs. A median of 4 surgeries were needed in case the ETV failed. (Table 2).

### Shunt revisions

In 13 of 35 shunted infants (37.1%), shunt revisions had to be done with an average of 1.8 revisions per child (range 1 to 5 revisions). Malfunction resulted from proximal or distal occlusion, disconnection, or valve dysfunction. On average, a shunt revision was necessary 112 days (range 1 to 635 days) after last performed procedure.

No ETV revisions have been performed. In 12 ETV treated patients (86%) CSF flow over the ostomy was detected in postoperative MRI studies. Two patients (14%) did not undergo postoperative MRI studies.

### Complications

Table 3 presents a percentage of complications within 30 days of a VAD, VPS or ETV procedure in relation to all performed procedures (54 VAD placements, 58 VPS and 16 ETVs). Most complications were seen in VAD interventions consisting of 3 CSF wound leakages (defined as CSF leakage through the skin) (5.6%), 2 VAD aspirations induced small periventricular white matter hemorrhage (3.7%), 1 superficial head wound infection (1.9%) and 1 candida meningitis/ventriculitis (1.9%). In the VPS group, 2 complications occurred within 30 days (1 CSF wound leakage and 1 superficial head wound infection) (both 1.7%). In the ETV group, 1 complication was observed (s. Aureus infection) (6.3%). All infections recovered well after antibiotic treatment.

**Table 3:** Complications rate within 30 days post-op (in percentage)

Complication < 30 days	VAD	VPS	ETV
CSF wound leakage	5.6	1.7	0
Hemorrhage	3.7	0	0
Wound infection	1.9	1.7	0
Infection (meningitis/ventriculitis)	1.9	0	6.3
Total	13.0	3.4	6.3

*VAD* ventricular access device, *VPS* ventriculoperitoneal shunt, *ETV* endoscopic third ventriculostomy, *CSF* cerebrospinal fluid

Regarding complications within 24 h, they only occurred in the VPS group. No complications occurred in either the VAD placements or the ETV group. The 2 complications in the 58 performed VPS surgeries consisted of CSF wound leakages (3.4%).

### ETV versus VPS

Regarding patient characteristics, no significant differences were found between both procedures, but the follow-up period for shunted patients tended to be longer (*p* = 0.09). Baseline values of treatments including age at time of VAD placement, VAD aspiration period and age at the time of permanent treatment showed no significant differences. Group sizes differed with twice as many patients in the VPS group. In addition, fewer premature infants were present in the ETV group. The etiology of preterm infants who received an ETV consisted of 5 IVH with stenosis, 2 aqueduct stenosis and 1 subarachnoidal cyst.

Forty of the 42 patients who received permanent treatment were under 5 months of age (32 patients were under 3 months of age). One patient was permanently treated when between 5 and 6 months corrected age. This patient first received a VAD and remained puncture independent after approximately 35 VAD aspiration days. At later follow-up, the patient presented with an enlarged head circumference with symptoms of increased ICP and subsequently required permanent hydrocephalus treatment.

Over the study period, there was a tendency of a higher failure rate in the ETV group compared to the VPS group (50% vs 32.1%, no significant difference). Time to failure was similar.

Table 4 shows ventricular measurements and CSF values within one week before permanent treatment. Concerning ventricular measurements, no significant differences were seen between both procedures. In the CSF, no significant differences were found in erythrocyte count or total protein between VPS or ETV treated patients.

**Table 4:** Ventricular measurements and CSF values within one week before permanent treatment

	Patients with VPS	Patients with ETV	*p* Value (95% CI)
VI in mm, mean (SD)			
Left VI	19.8 (5.7)	18.1 (4.5)	0.38 (−2.1 – 5.5)
Right VI	20.9 (7.0)	19.0 (3.9)	0.39 (−2.5 – 6.2)
AHW in mm, mean (SD)			
Left AHW	14.8 (6.5)	12.4 (8.2)	0.34 (−2.6 – 7.4)
Right AHW	15.5 (9.7)	13.0 (6.6)	0.44 (−3.8 – 8.6)
TOD in mm, mean (SD)			
Left TOD	36.7 (12.3)	34.1 (10.7)	0.54 (−5.9 – 11.0)
Right TOD	37.1 (13.8)	35.1 (10.4)	0.67 (−7.1 – 11.0)
CSF values, mean (SD)			
Erythrocytes (× 10^6^/L)	55 (131)	143 (237)	0.18 (−218 – 42)
Proteins (g/L)	0.83 (0.30)	0.69 (0.47)	0.32 (−0.14 – 0.41)

Missing data: left VI/AHW/TOD 5 missing (3 VPS, 2 ETV) (in two patients only measurements on the right ventricular system were known due to a cyst on the left side). Right VI/AHW 3 missing (1 VPS, 2 ETV), right TOD 4 missing (2 VPS, 2 ETV). Erythrocyte and protein value 9 missing (5 VPS, 4 ETV)

*VPS* ventriculoperitoneal shunt, *ETV* endoscopic third ventriculostomy, *CI* confidence interval, *SD* standard deviation, *VI* ventricular index, *AHW* anterior horn width, *TOD* thalamo-occipital distance, *mm* millimeters, *CSF* cerebrospinal fluid, *L* liter, *g* gram

### ETV success versus failure

Table 5 shows patients’ characteristics of patients treated with ETV as a first permanent treatment. The ETV was successful in 7 patients with hydrocephalus based on stenosis (whether or not combined with an IVH component). However, the ETV proved unsuccessful in 5 patients with congenital stenosis, as well as in one patient with an interhemispheric cyst and one with an infection (candida meningitis). The median ETVSS in failed and both successful ETVs concerns 40.

**Table 5:** Patients’ characteristics of patients initially treated with ETV

	ETV failed	ETV successful	*p* value (95% CI)
Total patients, *n* (%)	7 (50%)	7 (50%)	
Birth weight in grams, mean (SD)	2505 (991)	2466 (1234)	0.95 (−1343 – 1264)
Premature, *n* (%)	5 (71.4%)	3 (42.9%)	
Weeks of gestation, mean (SD)	35 (5)	35 (6)	0.95 (−7 – 6)
age at time of VAD placement in days, mean (SD)	27 (44)	23 (19)	0.84 (−48 – 40)
CSF aspiration via VAD in days, mean (SD)	40 (12)	38 (15)	0.81 (−21 – 17)
Follow-up period in days, mean (SD)	943 (272)	944 (359)	1.00 (−372 – 371)
Treatment age in days, mean (SD)	62 (38)	92 (76)	0.38 (−41 – 100)
ETVSS, median (IQR)	40 (30–40)	40 (40–40)	

*ETV* endoscopic third ventriculostomy, *CI* confidence interval, *SD* standard deviation, *VAD* ventricular access device, *CSF* cerebrospinal fluid, *ETVSS* endoscopic third ventriculostomy success score

In Table 6, greater differences were seen in the ventricular measurements between failed and successful ETVs, with generally higher values of the VI, AHW and TOD both left and right being measured in patients with a failed ETV. Erythrocyte counts and total proteins in the CSF in both failed and successful ETVs were similar.

**Table 6:** Ventricular measurements and CSF values of failed and successful ETV treated patients

	ETV failed	ETV successful	*p* Value (95%CI)
VI in mm, mean (SD)			
Left VI	19.8 (5.0)	15.6 (2.1)	0.11 (−9.6 – 1.1)
Right VI	20.9 (4.3)	16.4 (0.9)	0.05 (−8.9 – −0,1)
AHW in mm, mean (SD)			
Left AHW	16.4 (8.3)	6.8 (3.3)	0.04 (−18.5 – −0.8)
Right AHW	15.6 (7.5)	9.4 (2.9)	0.11 (−14.2 – 1.7)
TOD in mm, mean (SD)			
Left TOD	38.3 (10.9)	28.3 (8.1)	0.11 (−22.9 – 2.9)
Right TOD	39.5 (8.6)	29.0 (10.2)	0.08 (−22.6 – 1.6)
CSF values, mean (SD)			
Erythrocytes (× 10^6^/L)	154 (288)	125 (171)	0.86 (−402 – 344)
Proteins (g/L)	0.78 (0.48)	0.55 (0.48)	0.48 (−0.94 – 0.48)

Missing data: failed ETV 1 missing (erythrocyte and protein value of 1 patient). Successful ETV 5 missing (ventricular measurements of 2 patients, erythrocyte and protein values of 3 patients)

*ETV* endoscopic third ventriculostomy, *CI* confidence interval, *SD* standard deviation, *VI* ventricular index, *AHW* anterior horn width, *TOD* thalamo-occipital distance, *mm* millimeters, *CSF* cerebrospinal fluid, *L* liter, *g* gram

### Developmental follow-up

During follow-up, the patient’s developmental functioning was assessed using the BSID-III-NL assessment tool. This tool includes cognitive, motor, language, socio-emotional and adaptive behavior scales [21, 22]. The BSID-III-NL is designed to measure the developmental functioning of infants and toddlers in the Netherlands and is used to identify possible developmental delays. This BSID is standard care in extremely premature infants.

Table 7 presents the BSID-III-NL results of 23 patients. Results were not reported for the remaining 31 patients. Due to young age, 12 patients have not yet received observation in developmental functioning as described by the BSID-III-NL assessment tool. Nineteen patients experienced follow-up issues during COVID. Generally, an average/above average developmental functioning of cognitive ability and/or fine motor skills was identified in the 23 patients. Underdeveloped motor skills were especially identified in our two spina bifida patients with Chiari malformation.

**Table 7:** Follow-up using the BSID-III-NL assessment tool (*n* = 23)

	Total (percentage)	VAD only	VPS successful	VPS failed	ETV successful	ETV failed
Cognitive ability						
Average / above average	14 (87.5%)	2	7	2	1	2
Below average	2 (12.5%)	-	1	-	1	-
Fine motor skills						
Average / above average	9 (52.9%)	2	4	1	1	1
Below average	8 (47.1%)	2	2	1	2	1
Gross motor skills						
Average / above average	5 (41.7%)	2	-	2	-	1
Below average	7 (58.3%)	1	3	1	1	1
Linguistic problems						
Yes	2 (40%)	-	-	1	-	1
No	3 (60%)	2	1	-	-	-
Behavior problems						
Yes	4 (40%)	2	2	-	-	-
No	6 (60%)	-	2	2	2	-

Missing data: 31 patients (8 VAD only, 14 VPS, 9 ETV)

*BSID-III-NL Bayley Scales of Infant and Toddler Development-Third Edition-Dutch version*

## Discussion

In our study, in addition to the most used permanent treatment in hydrocephalic patients in the neonatal period, i.e. VPS placement, an ETV has shown to be a successful treatment option for hydrocephalus in well-selected very young infants.

Managing hydrocephalus in infants of less than 6 months of age, remains challenging with relatively high rates of surgery, shunt dependency and complications. Shunt operations are related to long-term complications, especially in premature newborns and young infants, high rates of complications have been reported [1, 8, 9]. With the development of endoscopic techniques, ETV may represent an important alternative to shunting in children with hydrocephalus. However, high ETV failure rates (55.3% – 83.5%) have been reported in neonates and young infants less than 6 months of age [2, 3, 7, 9–14]. In addition to these treatment options, neuro-endoscopic lavage under 3 months of age has gained interest in the treatment of hydrocephalus based on IVH. This procedure clears ventricular blood products and reduces the protein load. According to recent studies, neuro-endoscopic lavage might be a safe and effective treatment for neonatal IVH [23–26]. However, clear evidence regarding the reduction of shunt dependency and improving shunt survival for those requiring CSF diversion has not been reported yet. Moreover, after performing neuro-endoscopic lavage in young patients with IVH-related hydrocephalus, the majority of patients remain shunt dependent without a reduction in complications [25–27]. Also, cauterizing the choroid plexus in lateral ventricles might influence the success rate of ETV. The CSF production might reduce while waiting for absorption pathways to mature [28–30]. However, limited data is available on the success rates in very young infants [28–30].

This study cohort consisted of patients with diagnosed hydrocephalus in the neonatal period. The VPS group was larger than the ETV group, as ETV was only indicated in hydrocephalic neonates with an obstructive component. Despite differences in group size, patient characteristics of permanently treated groups were very similar. In our study group, a 50% ETV success rate seems to be higher than reported in the limited available studies, ranging from 16.5% to 44.7% [2, 3, 7, 9–14]. In patients between 0 and 3 months of age, the success rate is reported around 25%, and in patients between 3 and 6 months around 40% [2, 9, 13, 14]. Placing a VAD with subsequent CSF aspiration prior to thulium laser-assisted ETV in selected patients with an obstructive CSF pathway component may explain our study cohort's relatively low failure rate.

Predisposing patient factors that contribute to the ETV success score correspond to patient characteristics of our successful ETV patients. Patients with failed ETV tended to be younger (p = 0.38), which is equivalent to results presented by Kulkarni et al. [10]. Also, Koch et al. advocate that these infants, compared to older patients, have a higher propensity to form new arachnoid membranes which might be a cause of lower ETV success scores in younger patients [13]. According to Rahman et al., CSF infection or high proteins in CSF are predisposing factors that cause ETV failure [31]. Also, it is assumed that young infants have lower capacity for CSF absorption due to immature arachnoid granulation which requires a higher-pressure gradient for absorption [13, 32]. Therefore, the absorptive function of CSF through the subarachnoid space seems to be important and affects the success of the ETV [32]. Causes of hydrocephalus such as hemorrhage or infection can both lead to dysfunction of the subarachnoid space with a decrease in the absorption of CSF. Compared to aqueduct stenosis, hemorrhage or infection are less likely to be successfully treated with ETV [32]. It is suggested that the presence of growth factors in the CSF of young infants may result in stenosis of the ostomy [32]. In our cohort, no ETV ostomy closure was detected in postoperative MRI studies. This might be related to our center's hydrocephalus management strategy and/or ETV technique after a period of CSF tapping from the VAD.

Apart from these known factors that contribute to the success score of ETV, the ventricular measurements prior to ETV also seemed to influence the ETV success score in our cohort. Although the number of patients is small, lower values of ventricular measurements tended to be related to a higher ETV success score. A higher ventricular volume could have resulted from compression of the subarachnoid CSF spaces with a reducing effect of reabsorption of CSF. This could explain why there may be a higher rate of ETV failure in patients with large ventricular volumes, one week before permanent treatment.

Even though our study group had missing data, no differences were found in follow-up between the VPS and ETV treated patients using the BSID-III-NL assessment tool. According to Kulkarni et al., long-term outcomes of infants with symptomatic hydrocephalus from aqueduct stenosis treated with ETV or shunt suggest a high overall health status and quality of life and showed no significant difference between those treated initially with ETV or shunt [33].

The selection in this cohort is based on the VAD placement with punctures pre-operatively and performing an MRI prior to permanent treatment to differentiate between obstructive components in the CSF pathway versus communicating hydrocephalus. When obstructive imaging characteristics were seen, both VPS and ETV as permanent treatment were discussed in detail with the parents and a shared decision on surgical treatment was made. A shunt placement was done in a communicating hydrocephalus.

Although this study is of retrospective nature, performed at a single university hospital with a limited cohort of hydrocephalus patients, VAD CSF tapping, followed by permanent treatment showed relatively high success rates of our treatment strategies. In selected hydrocephalic infants below 3 months of age with an obstructive component in the CSF pathway, a thulium laser-assisted ETV appeared to be a valuable permanent treatment option. Relatively lower ventricular volumes prior to ETV tended to contribute to this relatively high ETV success rate. More longer-term research, preferably in a randomized trial setup, integrating motor and neurocognitive outcome data, is desirable to study the optimal management of hydrocephalus in this young patient group.

## Conclusion

Managing hydrocephalus in very young infants remains challenging regarding surgical strategy, reducing shunt dependency and decreasing current complication rates. In addition to VPS placements, an ETV has shown to be a successful treatment option for hydrocephalus in well-selected very young infants.

## Data Availability

No datasets were generated or analysed during the current study.
